# Association between serum level soluble programmed cell death ligand 1 and prognosis in patients with non‐small cell lung cancer treated with anti‐PD‐1 antibody

**DOI:** 10.1111/1759-7714.13721

**Published:** 2020-10-27

**Authors:** Shuji Murakami, Ryota Shibaki, Yuji Matsumoto, Tatsuya Yoshida, Yasushi Goto, Shintaro Kanda, Hidehito Horinouchi, Yutaka Fujiwara, Noboru Yamamoto, Yuichiro Ohe

**Affiliations:** ^1^ Department of Thoracic Oncology National Cancer Center Hospital Tokyo Japan

**Keywords:** Anti‐PD‐1 antibody, non‐small cell lung cancer, PD‐L1 TPS, soluble PD‐L1

## Abstract

**Background:**

Programmed cell death ligand 1 (PD‐L1) is known to have soluble forms aside from its membrane‐bound forms. The aim of this study was to evaluate the predictive and prognostic values of serum soluble PD‐L1 (sPD‐L1) in patients with non‐small cell lung cancer (NSCLC) who were treated with anti‐PD‐1 antibody.

**Methods:**

A total of 233 patients were enrolled in this study. We assessed the level of serum sPD‐L1 before anti‐PD‐1 antibody treatment (pembrolizumab or nivolumab) and evaluated the correlation with PD‐L1 expression on tumor cells, the response to anti‐PD‐1 antibody treatment, and patient outcome.

**Results:**

The median serum sPD‐L1 concentration was 67.7 (range, 25 to 223) pg/mL. A weak correlation between serum sPD‐L1 and tumor PD‐L1 expression was observed. The disease control rate in the high sPD‐L1 group (≥90 pg/mL) was significantly lower than that in the low sPD‐L1 group (<90 pg/mL) (37% vs. 57%, *P* = 0.0158). The progression‐free survival (PFS) and overall survival (OS) in the high sPD‐L1 group were significantly shorter than those in the low sPD‐L1 group (median PFS, 57 days vs. 177 days, *P* = 0.011; median OS, 182 days vs. not reached, *P* < 0.001). The high level of serum sPD‐L1 was independently associated with a shorter PFS (hazard ratio [HR], 1.910; *P* = 0.061) and OS (HR, 2.073; *P* = 0.034) in multivariate analysis.

**Conclusions:**

The serum sPD‐L1 level, which was only weakly correlated with the tumor PD‐L1 expression level, was an independent predictive and prognostic biomarker for NSCLC patients receiving anti‐PD‐1 antibody.

**Key points:**

**Significant findings of the study:**

The disease control rate in the high sPD‐L1 group was significantly lower than that in the low sPD‐L1 group. The progression‐free survival (PFS) and overall survival (OS) in the high sPD‐L1 group were significantly shorter than those in the low sPD‐L1 group. The high level of serum sPD‐L1 was independently associated with a shorter PFS and OS in multivariate analysis.

**What this study adds:**

This study demonstrated that serum sPD‐L1 level was an independent predictive and prognostic biomarker for NSCLC patients receiving anti‐PD‐1 antibody.

## Graphical abstract

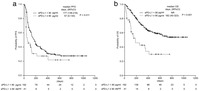

The PFS and OS of the high sPD‐L1 group were significantly shorter than those of the low sPD‐L1 group. The high level of serum sPD‐L1 was independently associated with a shorter PFS and OS.

## Introduction

Patients with advanced non‐small cell lung cancer (NSCLC) continue to have a poor prognosis. Platinum‐based chemotherapy for untreated advanced NSCLC still has a response rate of 20%–40% and confers a median survival period of about 12 months.^1^, ^2^ The discovery of driver mutations and the development of molecular targeted therapy for NSCLC have led to a paradigm shift in the treatment of advanced NSCLC.^3^ However, the clinical benefits are limited to patients with driver mutations.^4^, ^5^

In recent years, immune checkpoint inhibitors (ICIs) targeting the programmed death protein 1/programmed death ligand 1 (PD‐1/PD‐L1) pathway have shown a promising therapeutic effect against NSCLC, especially against tumors without driver mutations. In NSCLC, several anti‐PD‐1/PD‐L1 antibodies have been studied in several treatment settings, such as first‐line, second‐line, and maintenance.^6^, ^7^, ^8^, ^9^, ^10^, ^11^ In the second‐line setting, the use of anti‐PD‐1/PD‐L1 antibody actually improved the progression‐free survival (PFS) and overall survival (OS) periods, compared with chemotherapy.^7^, ^9^, ^10^, ^11^ However, the response rate remains at about 20% among advanced NSCLC patients receiving PD‐1/PD‐L1 antibody in unselected patients. PD‐L1 expressed on the tumor cells binds to PD‐1 receptors on activated T cells, which leads to the deactivation of cytotoxic T cells.^12^, ^13^ Blockade of the PD‐1/PD‐L1 pathway reactivates cytotoxic T cells and has been shown to produce unprecedented durable therapeutic responses.^14^, ^15^ Therefore, PD‐L1 expression on tumor cells has been defined as a predictive biomarker based on clinical trials. In nivolumab trials, tumor samples were categorized as positive when staining of the tumor‐cell membrane was observed at levels of 1%, 5%, or 10% of the cells. In previously treated patients with advanced nonsquamous NSCLC, nivolumab conferred higher objective response rates in the groups of patients whose tumors exhibited PD‐L1 expression levels of >1%, >5%, and >10%, but not in patients with PD‐L1 expression in <1% of their tumor cells (31% for the >1% group and 12% for the <1% group). However, in previously treated patients with advanced squamous NSCLC, PD‐L1 expression did not affect the efficacy of nivolumab, with a response rate of 17% for patients with a PD‐L1 expression ≥1% and for those with a PD‐L1 expression <1%.

In a pembrolizumab trial, the response rate for patients with a PD‐L1 tumor proportion score (TPS) of 50% or higher was 45.2%, compared with 16.5% in patients with a PD‐L1 TPS of 1%–49% and 10.7% in PD‐L1‐negative patients.^8^ Moreover, among untreated advanced NSCLC patients who were selected based on a PD‐L1 expression level of ≥50% on tumor cells, treatment with anti‐PD‐1 antibody (pembrolizumab) conferred a higher response rate of about 45% and a longer PFS and OS, compared with platinum‐based chemotherapy.^6^ Although the overall trend was a higher response for anti‐PD‐1/PD‐L1 antibody in PD‐L1‐positive patients, even if PD‐L1 expression was strongly positive, some patients exhibited disease progression immediately after treatment. Therefore, the expression of PD‐L1 on tumor cells is insufficient as a biomarker, and additional biomarkers are required to evaluate not only the biological characteristics of tumor cells, but also the immunological characteristics of patients.

Recently, a soluble form of PD‐L1 (sPD‐L1) has been detected in the peripheral blood of cancer patients.^16^, ^17^, ^18^, ^19^, ^20^ Serum sPD‐L1 can bind to PD‐1 receptors and may play an important role in immunoregulation.^21^ The sPD‐L1 level has been reported to be an adverse prognostic marker in several malignancies.^16^, ^17^, ^18^, ^20^, ^22^, ^23^ While the relationship between sPD‐L1 and PD‐L1 expression on tumor cells is intriguing, the correlation varies depending on the cancer type.^16^, ^24^ The relationship between the serum sPD‐L1 level and PD‐L1 expression on tumor cells and the prognostic value of serum sPD‐L1 in patients with advanced NSCLC also remain unknown.

In this study, we measured the pretreatment serum sPD‐L1 level in patients with advanced NSCLC who received anti‐PD‐1 antibody and assessed the relationship between the serum sPD‐L1 level and the clinical characteristics, PD‐L1 expression on tumor cells, the response to anti‐PD‐1 antibody, and patient outcome.

## Methods

### Patients and study design

Patients with advanced or recurrent NSCLC who received nivolumab or pembrolizumab as a first‐line to third‐line treatment between 1 December 2015, and 31 March 2018, at the National Cancer Center Hospital (Tokyo, Japan) were eligible for inclusion in this study. The end of the follow‐up period was 28 December 2018. Patients were excluded if they had insufficient serum samples available from before the start of treatment with anti‐PD‐1 antibody. We retrospectively reviewed the medical records of patients and evaluated the patient characteristics, laboratory findings for C‐reactive protein (CRP) and soluble PD‐L1, serum interferon gamma (IFN‐gamma), PD‐L1 expression on tumor cells, and outcome.

### Assessments of PD‐L1 expression on tumor cells and serum sPD‐L1

Immunohistochemistry for PD‐L1 expression on the tumor cells was performed using the commercially available PD‐L1 immunohistochemistry 22C3 pharmDx assay (Dako North America). PD‐L1 protein expression on tumor cells was evaluated by comparing the corresponding Hematoxylin‐eosin stain sections to discriminate tumor cells from the other immune and stromal cells and the tumor proportion score (TPS) was defined as the percentage of at least 100 viable tumor cells showing partial or complete membrane staining. The PD‐L1 TPS was classified into three group: TPS lower than 1%, TPS of 1% to 49%, and TPS of 50% or higher. A PD‐L1 TPS of 50% or higher was classified as strongly positive.

Blood serum samples were obtained from each subject and stored at 4°C until the next day and subsequently stored at −20°C until further processing at the National Cancer Center Biobank (Tokyo, Japan). Serum samples from the patients who were eligible for this study were sent to a clinical laboratory for measurement of the sPD‐L1 and IFN‐gamma concentrations. The serum concentrations of sPD‐L1 were measured using a Human/Cynomolgus Monkey PD‐L1/B7‐H1 Quantikine Enzyme‐Linked Immunosorbent Assay (ELISA) kit (Catalog Number DB7H10). We defined the sPD‐L1 cutoff value as 90 pg/mL, which was the mean value plus two standard deviations (62.5 + 27.4 pg/mL) of the levels in healthy volunteers, as obtained from the catalog data for the ELISA kit. The patients were divided into two groups according to this sPD‐L1 cutoff value. The IFN gamma level was measured using a commercial Human IFN gamma Platinum ELISA kit (Catalog Number BMS228/BMS228TEN).

### Statistical analysis

Differences in serum sPD‐L1 concentrations were tested using the Student *t*‐test. The baseline characteristics were compared between patients with high serum PD‐L1 levels and those with low PD‐L1 levels using a Chi‐square test and the Student *t*‐test. Pearson correlation analysis was used to analyze the correlations between the sPD‐L1 concentration and the PD‐L1 TPS on tumor cells. The objective response rates and the disease control rates were compared using the chi‐square test. PFS was defined as the period between the date of the first dose of anti‐PD‐1 antibody treatment and the date of clinical or radiographic disease progression or death from any cause. OS was defined as the period between the date of the first dose of anti‐PD‐1 antibody treatment and the date of death from any cause. The survival curves were calculated and drawn using the Kaplan‐Meier method, and groups were compared using the log‐rank statistic. Univariate and multivariate prognostic analyses of PFS and OS were performed using the Cox‐regression model. The results were considered statistically significant when *P* < 0.05. All the statistical analyses were performed using SPSS 19.0 (IBM, Armonk, NY, USA).

## Results

### Patient characteristics

A total of 233 patients with advanced or recurrent NSCLC who were started on anti‐PD‐1 antibody (pembrolizumab or nivolumab) were enrolled in this study. The patient characteristics are summarized in Table 1. The median age of the patients overall was 63 years (range: 30–84 years); 152 (65%) patients were male; 211 (91%) had a good performance status (PS) (0–1); 54 (23%) were never smokers; 52 (22%) had squamous cell carcinoma; and 37 (16%) had epidermal growth factor receptor (EGFR) mutation. PD‐L1 testing for the tumors was performed in 156 (67%) patients, with PD‐L1 TPS <1% seen in 33 (14%) patients, 1% to 49% seen in 44 (19%) patients, and ≥50% seen in 79 (34%) patients. A total of 40 (17%) patients received pembrolizumab as the first‐line therapy. The median serum sPD‐L1 concentration was 67.7 (range: 25–223) pg/mL. Using a cutoff value of 90 pg/mL, 41 (18%) patients were classified into the high‐sPD‐L1 group (≥90 pg/mL). The median CRP concentration was 1.31 (range: 0–28.3) mg/dL. Serum IFN‐gamma was undetectable (<1.56 pg/mL) in 195 (83%) patients.

**Table 1:** Patient characteristics stratified according to soluble PD‐L1 expression

		sPD‐L1 < 90 pg/mL	sPD‐L1 ≥ 90 pg/ mL	
Characteristics	*N* = 233	(*N* = 192)	(*N* = 41)	*P*‐value
Age				
Median (range)	63 (30–84)	63 (30–84)	64 (36–79)	0.458
≥75 years	30 (13%)	25 (13%)	5 (12%)	
Sex				0.416
Female	81 (35%)	69 (36%)	12 (30%)	
Male	152 (65%)	123 (64%)	29 (70%)	
ECOG PS				0.107
0–1	211 (91%)	177 (92%)	34 (83%)	
2	22 (9%)	15 (8%)	7 (17%)	
Smoking status				0.540
Never	54 (23%)	46 (24%)	8 (20%)	
Current to former smoker	179 (77%)	146 (76%)	33 (80%)	
Histology				0.713
Squamous	52 (22%)	42 (22%)	10 (24%)	
Nonsquamous	181 (78%)	150 (78%)	31 (76%)	
Brain metastasis				0.055
Absent	180 (77%)	153 (80%)	27 (66%)	
Present	53 (23%)	39 (30%)	14 (34%)	
Liver metastasis				0.015
Absent	197 (85%)	169 (88%)	28 (68%)	
Present	36 (15%)	23 (12%)	13 (32%)	
Pulmonary metastasis				0.883
Absent	167 (72%)	138 (72%)	29 (71%)	
Present	66 (28%)	54 (28%)	12 (29%)	
EGFR				0.264
Wild‐type	135 (58%)	116 (60%)	19 (46%)	
Mutation	37 (16%)	29 (15%)	8 (20%)	
PD‐L1 TPS				0.163
< 1%	33 (14%)	30 (16%)	3 (7%)	
1% to 49%	44 (19%)	38 (20%)	6 (15%)	
≥ 50%	79 (34%)	61 (32%)	18 (44%)	
Treatment‐line of anti‐PD‐1				0.636
First	40 (17%)	34 (18%)	6 (15%)	
Second or third	193 (83%)	158 (82%)	35 (85%)	
Prior treatments				
Chemotherapy	193 (83%)	158 (82%)	35 (85%)	0.636
TKI	35 (15%)	27 (14%)	8 (20%)	0.375
Thoracic radiotherapy	90 (39%)	79 (41%)	11 (27%)	0.087
CRP (mg/dL)				
Median (range)	1.31 (0–28.3)	0.74 (0.01–19.4)	6.79 (0–28.3)	<0.001
< 1.31	114 (49%)	110 (57%)	4 (10%)	
≥ 1.31	115 (49%)	78 (41%)	37 (90%)	
IFN‐gamma (pg/mL)				
Undetectable (<1.56)	195 (83%)	167 (87%)	28 (68%)	0.002
Detectable, median (range)	4.295 (1.87–886)	5.59 (1.87–886)	3.61 (2.09–89)	
Serum sPD‐L1 (pg/mL)				
Median (range)	67.7 (25–223)	62.25 (25–89.5)	108 (90.1–223)	
No. of cases with progression or relapse	158 (68%)	129 (67%)	29 (71%)	
No. of deaths	97 (42%)	73 (38%)	24 (59%)	

CRP, C‐reactive protein; ECOG PS, Eastern Cooperative Oncology Group performance status; EGFR, epidermal growth factor receptor; IFN, interferon; No., number; sPD‐L1, soluble programmed death ligand 1; TPS, tumor proportion score.

### Correlation between soluble PD‐L1 and clinicopathological characteristics

**Figure 1:**
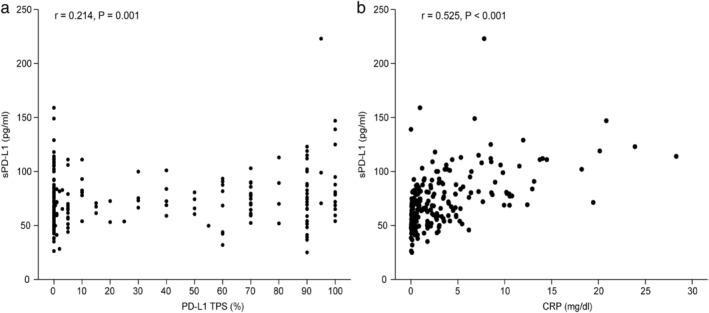
Linear regression analysis of serum soluble PD‐L1 (sPD‐L1) level and (**a**) PD‐L1 tumor proportion score (TPS) on tumor cells and (**b**) serum CRP.

The mean serum sPD‐L1 concentration was 64.4 ± 17.5 pg/mL in patients with a PD‐L1 TPS of <1%, 70.6 ± 18.0 pg/mL in patients with a PD‐L1 TPS of 1% to 49%, and 77.7 ± 28.9 pg/mL in patients with a PD‐L1 TPS ≥50%; this difference was statistically significant (*P* = 0.0101). A statistically significant but weak linear correlation between the serum sPD‐L1 concentration and the tumor PD‐L1 expression was seen (*r* = 0.214, *P* = 0.001) (Fig 1a). No significant correlation was found in the 40 patients who received first‐line treatment with anti‐PD‐1 antibody (*r* = 0.160, *P* = 0.346).

The mean serum sPD‐L1 concentration was 68.0. ± 22.6 pg/mL in females and 73.6 ± 25.9 pg/mL in males, 67.0 ± 21.8 pg/mL in never smokers and 72.8 ± 25.4 pg/mL in current or former smokers. It was 72.4 ± 23.3 pg/mL in patients with squamous cell carcinoma and 71.5 ± 25.4 pg/mL in patients with nonsquamous cell carcinoma, 70.3 ± 21.2 pg/mL in patients without brain metastasis and 76.5 ± 34.5 pg/mL in patients with brain metastasis, 72.2 ± 26.6 pg/mL in patients without pulmonary metastasis and 70.4 ± 20.2 pg/mL in patients with pulmonary metastasis, and 71.0 ± 25.4 pg/mL in patients with *EGFR* wild‐type and 69.4 ± 24.1 pg/mL in patients with an *EGFR* mutation; these values were not significantly different (*P* > 0.05). Meanwhile, the serum sPD‐L1 concentration was higher in patients with liver metastasis than in those without liver metastasis (80.9 ± 36.6 pg/mL vs. 70.0 ± 21.8 pg/mL, *P* = 0.015). No significant differences in patient characteristics, including age, sex, the Eastern Cooperative Oncology Group Performance Status Scale (ECOG PS), smoking history, histology, presence of *EGFR* mutation, presence of brain metastasis, presence of pulmonary metastasis, and serum IFN‐gamma level, were seen between patients with a high serum sPD‐L1 concentration and those with a low sPD‐L1 concentration. The presence of liver metastasis and the serum CRP level was significantly higher in the high sPD‐L1 group than in the low sPD‐L1 group (*P* < 0.001). The serum sPD‐L1 concentration was moderately correlated with the CRP level (*r* = 0.525, *P* < 0.001) (Fig 1b).

### Impact of serum sPD‐L1 level on efficacy of anti PD‐1 antibody and prognosis in NSCLC patients

**Figure 2:**
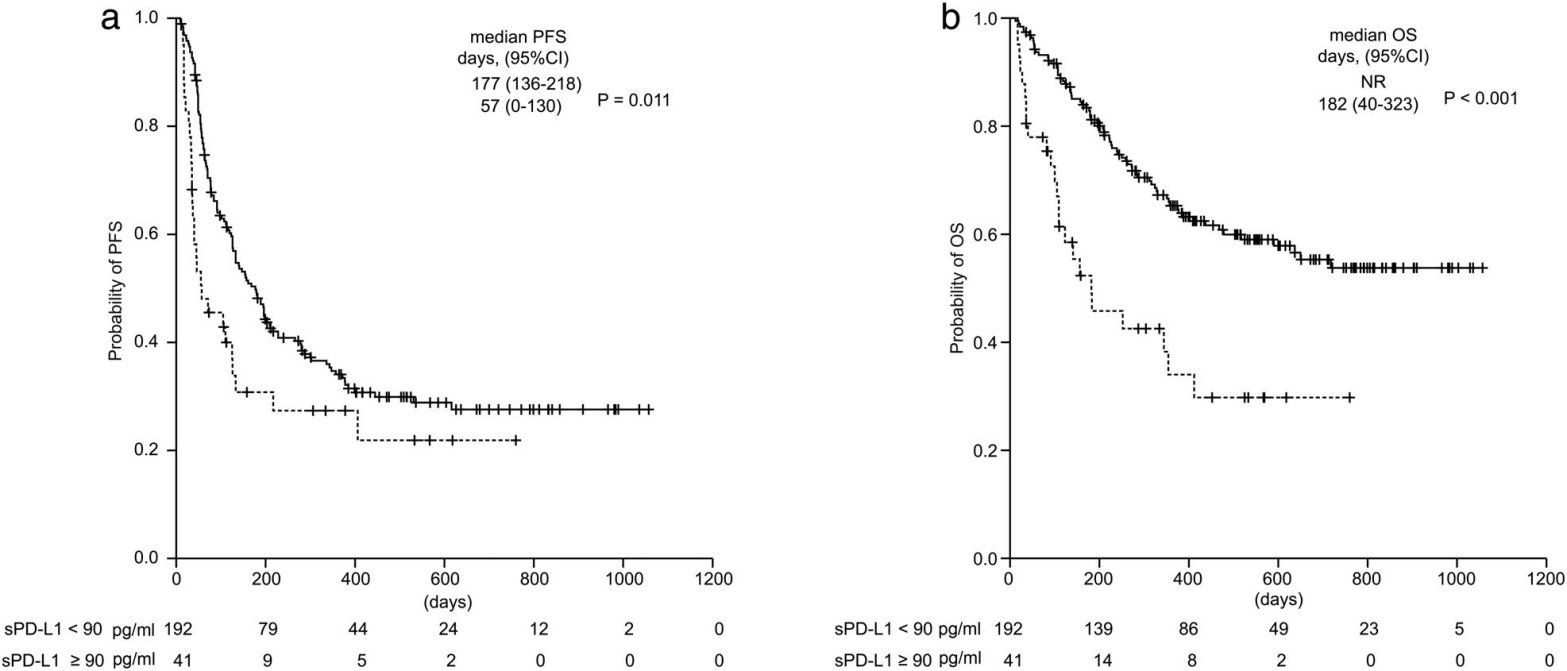
Kaplan‐Meier curves of (**a**) progression‐free survival and (**b**) overall survival for all the patients treated with anti‐PD‐1 antibody. (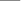) sPD‐L1 <90 pg/mL, and (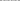) sPD‐L1 ≥90 pg/mL sPD‐L1, soluble PD‐L1; PFS, progression‐free survival; OS, overall survival; CI, confidence interval.

The overall response rate (ORR) was similar between patients in the high and low sPD‐L1 groups (ORR: 22% [95% CI: 19–31] vs. 24% [95% CI: 11–38]; *P* = 0.731), but the disease control rate (DCR; defined as a complete or partial response or stable disease) in the high sPD‐L1 group was significantly lower than that in the low sPD‐L1 group (DCR: 37% [95% CI: 22–53] vs. 57% [95% CI: 50–64], *P* = 0.0158) (Table 2). The PFS and OS of the high sPD‐L1 group were significantly shorter than those of the low sPD‐L1 group (median PFS: 57 days [95% CI: 0–130] vs. 177 days [95% CI: 126–218], *P* = 0.011; median OS: 182 days [95% CI: 40–323] vs. not reached days, *P* < 0.001) (Fig 2).

**Table 2:** Response to anti‐PD‐1 antibody

		sPD‐L1 < 90	sPD‐L1 ≥ 90	
Response	*N* = 233	(*N* = 192)	(*N* = 41)	*P*‐value
Complete response	0	0	0	
Partial response	56 (24%)	47 (24%)	9 (22%)	
Stable disease	69 (30%)	63 (32%)	6 (15%)	
Progressive disease	100 (43%)	78 (40%)	22 (54%)	
Not evaluable	8 (3%)	4 (2%)	4 (10%)	
ORR% (95% CI)	24% (19%–30%)	24% (11%–38%)	22% (19%–31%)	0.731
DCR% (95% CI)	54% (47%–60%)	57% (50%–64%)	37% (22%–53%)	0.0158

The disease control rate was defined as the percentage of patients with a complete or partial response or stable disease.

DCR, disease control rate; ORR, objective response rate; sPD‐L1, soluble programmed death ligand 1.

Covariates with significant PFS and OS differences in univariate analyses were subsequently entered into a multivariate analysis (Table 3). Strongly positive PD‐L1 expression on tumor cells (HR, 0.486 [95% CI: 0.277–0.852]; *P* = 0.001) and an sPD‐L1 concentration ≥90 pg/mL (HR, 1.910 [95% CI: 0.972–3.753]; *P* = 0.061) were independent predictors for PFS. Similarly, PS 2 (HR, 3.342 [95% CI: 1.674–6.670]; *P* < 0.001), Liver metastasis (HR, 2.099 [95% CI: 1.106–3.76]; *P* = 0.022), brain metastasis (HR, 2.406 [95% CI: 1.413–4.095]; *P* = 0.001), strongly positive PD‐L1 expression (HR, 0.621 [95% CI: 0.365–1.59]; *P* = 0.08), a CRP level ≥ 1.31 (HR, 2.259 [95% CI: 1.298–3.932]; *P* = 0.004), and an sPD‐L1 concentration ≥ 90 pg/mL (HR, 2.073 [95% CI: 1.056–4.066]; *P* = 0.034) were independent prognostic factors for OS.

**Table 3:** Cox proportional hazard regression analyses to assess the impact of clinicopathological variables on PFS and OS

	Univariate analysis		Multivariate analysis	
Variables	HR (95% CI)	*P*‐value	HR (95% CI)	*P*‐value
PFS				
PS (0–1/2)	1.951 (1.192–3.192)	0.008	1.719 (0.780–3.788)	0.179
Age (<75/≥75)	0.744 (0.455–1.217)	0.239	‐	
Sex (female/male)	0.706 (0.511–0.974)	0.034	0.765 (0.440–1.330)	0.342
Smoking status (never/current or former)	0.547 (0.385–0.777)	0.001	0.794 (0.404–1.560)	0.503
Pathology (SQ/nonSQ)	1.082 (0.742–1.577)	0.683	‐	
EGFR (wt/mt)	1.873 (1.235–2.841)	0.003	1.296 (0.664–2.531)	0.448
Liver metastasis (absent/present)	1.900 (1.261–2.864)	0.002	1.343 (0.664–2.715)	0.412
Brain metastasis (absent/present)	1.702 (1.201–2.412)	0.003	1.165 (0.678–2.002)	0.582
PD‐L1 TPS (<50%/≥50%)	0.572 (0.383–0.854)	0.006	0.486 (0.277–0.852)	0.001
PD‐L1 TPS (<1%/≥1%)	0.618 (0.392–0.974)	0.038	‐	
Treatment line (1/2–3)	1.795 (1.111–2.900)	0.017	1.383 (0.716–2669)	0.334
CRP (<1.31/≥1.31)	1.45 (1.058–1.9988)	0.021	1.429 (0.867–2.355)	0.162
sPD‐L1 (<90/≥90)	1.677 (1.119–2.512)	0.012	1.910 (0.972–3.753)	0.061
OS				
PS (0–1/2)	3.261 (1.897–5.604)	<0.001	3.342 (1.674–6.670)	<0.001
Age (<75/≥75)	0.784 (0.418–1.469)	0.477	‐	
Sex (female/male)	1.031 (0.677–1.570)	0.886	‐	
Smoking status (never/current or former)	0.908 (0.572–1.441)	0.908	‐	
Pathology (SQ/nonSQ)	0.865 (0.541–1.382)	0.865	‐	
EGFR (wt/mt)	0.998 (0.573–1.738)	0.998	‐	
Liver metastasis (absent/present)	2.747 (1.727–4.370)	<0.001	2.099 (1.106–3.760)	0.022
Brain metastasis (absent/present)	2.216 (1.450–3.389)	<0.001	2.406 (1.413–4.095)	0.001
PD‐L1 TPS (<50%/≥50%)	0.690 (0.417–1.142)	0.149	0.621 (0.365–1.059)	0.08
PD‐L1 TPS (<1%/≥1%)	0.654 (0.377–1.132)	0.129	‐	
Treatment line (1/2–3)	1.051 (0.747–1.479)	0.776	‐	
CRP (<1.31/≥1.31)	2.732 (1.791–4.170)	<0.001	2.259 (1.298–3.932)	0.004
sPD‐L1 (<90/≥90)	2.663 (1.671–4.245)	<0.001	2.073 (1.056–4.066)	0.034

CI, confidence interval; CRP, C‐reactive protein; HR, hazard ratio; OS, overall survival; PFS, progression‐free survival; SQ, squamous cell; PS, performance status; sPD‐L1, soluble programmed death ligand 1; TPS, tumor proportion score.

**Figure 3:**
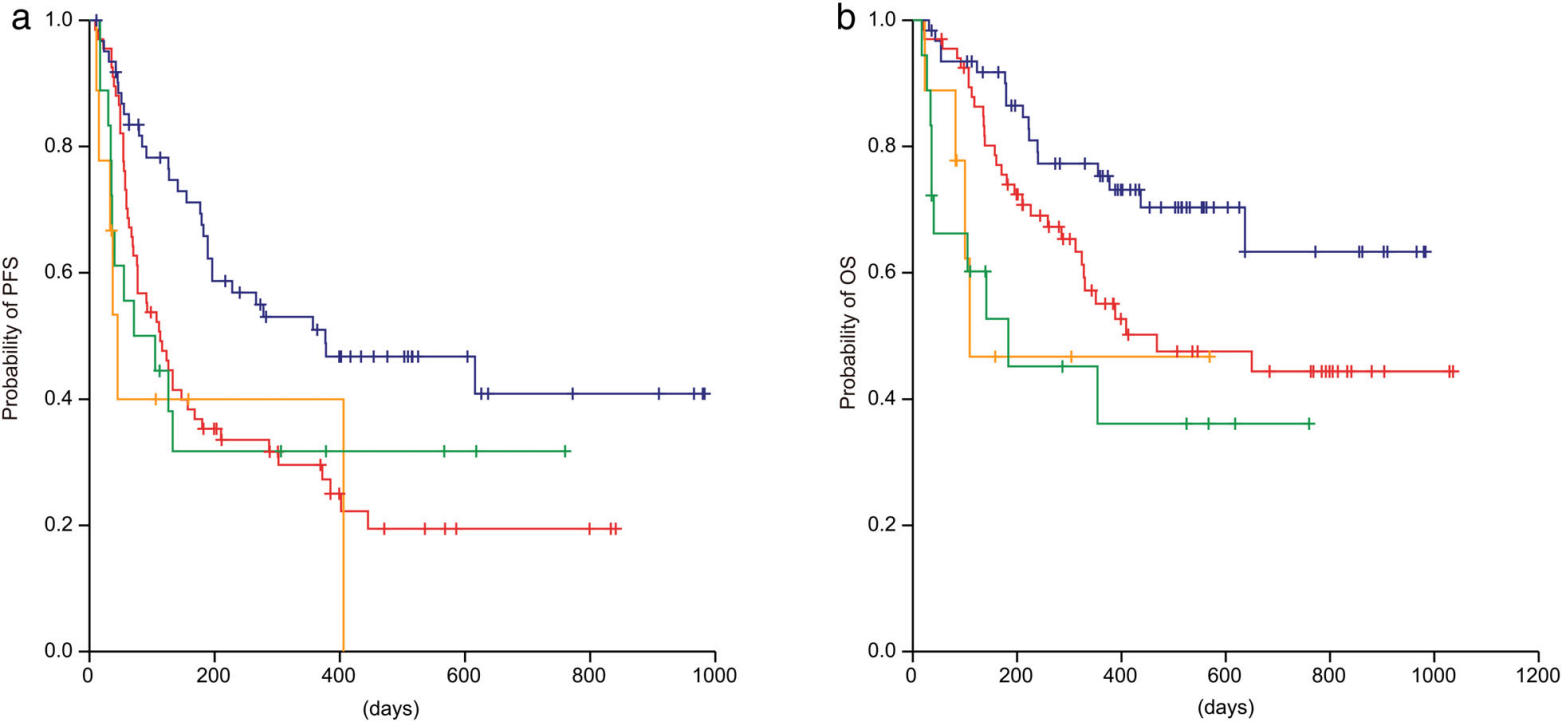
Kaplan‐Meier curves of (**a**) progression‐free survival and (**b**) overall survival among four groups: (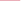) Group 1: PD‐L1<50% + sPD‐L1 < 90 pg/mL (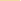) Group 2: PD‐L1<50% + sPD‐L1 ≥ 90 pg/mL (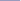) Group 3: PD‐L1≥50% + sPD‐L1 < 90 pg/mL (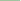) Group 4: PD‐L1≥50% + sPD‐L1 ≥ 90 pg/mL. sPD‐L1, soluble PD‐L1; PFS, progression‐free survival; OS, overall survival; CI, confidence interval; TPS, tumor proportion score.

An additional survival analysis was performed among patients who were classified into four groups according to PD‐L1 expression on tumors cells and the sPD‐L1 concentration: group 1, low levels of both PD‐L1 TPS and sPD‐L1; group 2, not strongly positive PD‐L1 but high sPD‐L1; group 3, strongly positive PD‐L1 but low sPD‐L1; and group 4, high levels of both PD‐L1 TPS and sPD‐L1. The patient characteristics of the four groups are summarized in Table 4. Among the patients with strongly positive PD‐L1 expression, the PFS and OS of the high sPD‐L1 group (group 4) was shorter than that of the patients in the low sPD‐L1 group (group 3) (median PFS: 71 [95% CI: 0–175] vs. 377 [95% CI: 56–698] days; median OS: 183 [95% CI: 0–441] vs. not reached days). Compared with groups 1 and 3, even in the low sPD‐L1 group, the PFS and OS of the patients with strongly positive PD‐L1 expression (group 1) were shorter (median PFS: 113 [95% CI: 74–152] days; median OS: 468 [95% CI: 85–851] days) than those of the patients without strongly positive PD‐L1 expression (group 3) (Fig 3).

**Table 4:** Patient characteristics of four groups

	Group 1	Group 2	Group 3	Group 4
PD‐L1 TPS	<50%	<50%	≥50%	≥50%
sPD‐L1 (pg/mL)	<90	≥90	<90	≥90
Characteristics	(*N* = 68)	(*N* = 9)	(*N* = 60)	(*N* = 18)
Age				
Median (range)	64 (30–84)	64 (36–79)	63.5 (34–85)	63 (48–770
≧75 years	6 (9%)	1 (11%)	12 (20%)	3 (17%)
Gender				
Female	25 (37%)	4 (44%)	19 (32%)	5 (28%)
Male	43 (63%)	5 (56%)	41 (68%)	13 (72%)
ECOG PS				
0–1	63 (93%)	7 (78%)	54 (90%)	15 (83%)
2	5 (7%)	2 (22%)	6 (10%)	3 (17%)
Smoking status				
Never	15 (22%)	3 (33%)	12 (20%)	2 (11%)
Current to former smoker	53 (78%)	6 (67%)	48 (80%)	16 (89%)
Histology				
Squamous	17 (25%)	3 (33%)	10 (17%)	2 (11%)
Nonsquamous	51 (75%)	6 (67%)	50 (83%)	16 (89%)
EGFR				
Wild‐type	44 (65%)	4 (44%)	41 (68%)	12 (67%)
Mutation	8 (12%)	1 (11%)	11 (18%)	3 (17%)
PD‐L1 TPS				
< 50%	68 (100%)	9 (100%)	0	0
≧ 50%	0	0	60 (100%)	18 (100%)
Treatment‐line				
1	5 (7%)	1 (11%)	26 (43%)	5 (28%)
2–3	63 (93%)	8 (89%)	34 (57%)	13 (72%)
CRP				
Median (range)	0.655 (0.01–19.44)	6.45 (0.31–14.49)	0.785 (0.04–12.41)	8.51 (1.19–23.89)
< 1.31	42 (62%)	1 (11%)	36 (60%)	2 (11%)
≧ 1.31	26 (38%)	8 (89%)	24 (40%)	16 (89%)
IFN‐gamma				
Undetectable (<1.56)	62 (91%)	7 (78%)	52 (87%)	12 (67%)
Detectable, median (range)	6.86 (2.84–886)	3.49 (2.97–4.01)	14.95 (2.88–150)	3.48 (2.67–89)
Serum sPD‐L1				
Median (range)	63.9 (28.3–87.1)	106(92.6–112)	69.25 (25–89.5)	107.5 (90.1–223)
No. of progression or relapse	50 (74%)	6 (67%)	30 (50%)	12 (67%)
No. of deaths	31 (46%)	4 (44%)	17 (28%)	10 (56%)

CRP, C‐reactive protein; ECOG PS, Eastern Cooperative Oncology Group performance status; EGFR, epidermal growth factor receptor; IFN, interferon; No., number; sPD‐L1, soluble programmed death ligand 1; TPS, tumor proportion score.

## Discussion

Although some previous studies have reported that a high serum sPD‐L1 level is associated with a poor prognosis in patients with several types of cancer,^20^, ^22^, ^23^, ^25^, ^26^ few reports have discussed the relationship between the serum sPD‐L1 level and the clinical outcomes of cancer patients receiving anti‐PD‐1 antibody. Therefore, the relationship between serum sPD‐L1 and PD‐L1 expression on tumor cells and the prognostic value of sPD‐L1 in patients with advanced NSCLC remain unknown. In the present study, we found that the serum sPD‐L1 concentration was weakly correlated in a linear manner with PD‐L1 expression on tumor cells and that a high serum sPD‐L1 level was a negative predictor of disease control using anti‐PD‐1 antibody (pembrolizumab or nivolumab) and an independent negative predictor of prognosis in advanced NSCLC patients receiving anti‐PD‐1 antibody (pembrolizumab or nivolumab). Indeed, even in patients with strongly positive PD‐L1 expression, the patients with a high sPD‐L1 level had a shorter PFS and OS than those with a low sPD‐L1 level.

PD‐L1 is commonly overexpressed on certain tumor cells.^12^ Furthermore, the PD‐1/PD‐L1 pathway is a critical mechanism of immune activation and plays an important role in immunological tolerance.^13^ However, PD‐L1 is widely expressed on the membranes of hematopoietic and nonhemopoietic cells other than cancer cells, such as B and T lymphocytes, dendric cells (DCs), macrophages, and vascular endothelial cells, etc. The expression of PD‐L1 is regulated by inflammatory cytokines, such as type 1 IFN, type 2 IFN (IFN‐gamma), and TNF‐α.^13^ Recently, PD‐L1 has been reported to have soluble forms aside from their membrane‐bound forms, increasing the complexity of the PD‐1/PD‐L1 pathway.^27^ sPD‐L1 is thought to be derived from cells expressing PD‐L1, making immune cells and tumor cells potential sources of sPD‐L1.^22^, ^28^, ^29^ The serum concentration of sPD‐L1 in cancer patients, including those with advanced NSCLC, were significantly upregulated, compared with those in healthy controls.^19^, ^26^ However, most previous studies have reported no association between PD‐L1 expression on tumor cells and the sPD‐L1 level in patients with diffuse large B‐cell lymphomas,^17^ renal cell carcinomas,^30^ or pancreatic cancer.^24^ Likewise, the expression of PD‐L1 on tumor cells was only weakly correlated with the serum sPD‐L1 level in patients with NSCLC in the present study. Which cells produce sPD‐L1 and how the production of sPD‐L1 is regulated are unclear. Moreover, the immunological significance of sPD‐L1 in cancer patients is not fully understood. A previous study reported that sPD‐L1 is mainly released from activated mature DCs, and sPD‐L1 released by activated mature DCs induced the apoptosis of CD4+ and CD8+ T cells.^28^ Moreover, another study reported that tumor cell‐derived sPD‐L1 can induce apoptosis in T cells.^29^ These findings suggest that sPD‐L1 has the potential to regulate immune homeostasis and to affect tumor immunity. Moreover, competition between sPD‐L1 and anti‐PD‐1 antibody for membranous PD‐1 binding on T lymphocytes may reduce the pharmacodynamic activity of anti‐PD‐1 antibody, potentially reducing the efficacy of this therapy. The serum sPD‐L1 level has been reported to be upregulated in patients with elevated markers of systemic inflammation, such as CRP, in hepatocellular carcinoma (HCC),^31^ gastric cancer,^18^ or pancreatic cancer.^24^ The present study also indicated that the serum CRP level was associated with the serum sPD‐L1 level. In a melanoma cell line, the sPD‐L1 level could be increased by coculturing the cells with pro‐inflammatory cytokines, such as IFN‐gamma.^25^ Serum IFN‐gamma was only detected in 17% of the patients in the present study. Therefore, we could not adequately evaluate the relationship between serum sPD‐L1 and pro‐inflammatory cytokines. The plasma sPD‐L1 level of NSCLC patients has been reported to not differ significantly according to age, sex, histological type, *EGFR* mutation status, smoking history.^23^ Likewise, the sPD‐L1 concentration was not affected by patient characteristics such as sex, smoking history, histology, or *EGFR* status in the present study. On the other hand, a previous study reported a trend toward an increase in the plasma sPD‐L1 level according to the number of metastatic sites in patients with NSCLC.^32^ Other studies have demonstrated that a higher serum sPD‐L1 level was observed in HCC and renal cell carcinoma patients with a larger tumor size and metastasis.^16^, ^33^ These findings suggest that the serum sPD‐L1 level varies depending on the tumor burden. All the patients enrolled in the present study had recurrent disease after definitive local treatment or advanced NSCLC, and some patients had already been treated with some type of systemic therapy; therefore, the relationship between the primary tumor size or the clinical stage and the sPD‐L1 level could not be evaluated. However, we found that a higher serum sPD‐L1 level was associated with the presence of liver metastasis, but not with the presence of brain metastasis or pulmonary metastasis. Considering the results of the present study and previous studies, the sPD‐L1 level might be influenced by the tumor burden and cancer‐induced inflammation.

The serum sPD‐L1 level has been reported to have prognostic value for several types of cancer.^20^, ^22^, ^23^, ^25^, ^26^ Indeed, a high serum sPD‐L1 level was reported to be associated with a worse prognosis than a low expression level in patients with advanced NSCLC (18.7 vs. 26.8 month, *P* < 0.001)^26^ and in patients with advanced lung cancer (13.0 vs. 20.4 months, *P* = 0.037).^23^ Moreover, some studies regarding the treatment response to cytotoxic chemotherapy without ICIs have reported that the serum sPD‐L1 level was a negative therapeutic biomarker in patients with multiple myeloma^25^ or lymphoma.^20^ From these findings, serum sPD‐L1 could be a poorer prognostic factor for NSCLC patients regardless of treatment. Therefore, the predictive value of the serum sPD‐L1 level in patients receiving anti‐PD‐1 antibody remains unknown for most cancer types.

Recently, many researchers have focused on the exploration of predictive biomarkers for the efficacy of ICIs, such as the tumor mutation burden, gene expression profiling, tumor‐infiltrated lymphocytes, and peripheral blood markers.^34^ A few reports have evaluated the predictive value of the serum sPD‐L1 level among patients receiving ICIs. In melanoma patients treated with ICIs including anti‐CTLA4‐antibody and anti‐PD‐1 antibody, high pretreatment levels of serum sPD‐L1 were associated with an increased likelihood of progressive disease in patients treated with ICIs.^34^ However, this previous study was relatively small with only 35 patients receiving anti‐PD‐1 antibody; therefore, the number of subjects was insufficient to evaluate the statistical difference. The present study showed that the DCR in the high serum sPD‐L1 group was significantly lower than that in the low serum sPD‐L1 group (37% vs. 57%). Moreover, a Cox regression analysis revealed that a higher sPD‐L1 level was a noteworthy independent prognostic factor of a lower PFS and OS. Of note, especially in patients with strong PD‐L1 expression on tumor cells, a high serum sPD‐L1 level was an independent negative predictor. Of course, these results may depend on the different sources of PD‐L1 and the different roles of the anti‐PD‐1/PD‐L1 pathway in the tumor environment and in host immune surveillance. A previous study indicated that an elevated serum CRP level was independently associated with a worse response and a shorter survival in patients treated with nivolumab.^35^ In the present study, a high serum CRP level was associated with a poorer PFS in a univariate analysis and a poorer OS in univariate and multivariate analyses, but was not significantly associated with PFS in a multivariate analysis. In cancer patients, elevated serum CRP levels are higher than those in healthy individuals and are generally associated with tumor burden, disease progression, a deteriorated physical status, and decreased survival.^36^, ^37^ According to these findings, an elevated serum CPR might have prognostic value. Recent studies have focused on the association between the change in the serum CRP level from baseline and the efficacy of ICIs,^38^, ^39^ but the predictive value of an elevated serum CPR level at baseline remains unclear.

In conclusion, we determined that the serum sPD‐L1 level was associated with the presence of liver metastasis and inflammatory markers such as CRP and was weakly correlated with tumor PD‐L1 expression. Furthermore, serum sPD‐L1 levels may be an independent predictive and prognostic biomarker for NSCLC patients receiving anti‐PD‐1 antibody (pembrolizumab or nivolumab).

## Disclosure

Y. Goto has served on speakers' bureaus for Ono Pharmaceutical, Bristol‐Myers Squibb, and MSD; and received research funding from Bristol‐Myers Squibb, and Ono Pharmaceutical. S. Kanda has received research funding from Ono Pharmaceutical; and received honoraria from Ono Pharmaceutical, and Bristol‐Myers Squibb. H. Horinouchi has received research funding from MSD, Bristol‐Myers Squibb, and Ono Pharmaceutical. Y. Fujiwara has received research funding from MSD, and Bristol‐Myers Squibb; and served on speakers' bureaus from MSD, Bristol‐Myers Squibb, and Ono Pharmaceutical. N. Yamamoto has received research funding from Bristol‐Myers Squibb, and Ono Pharmaceutical; and served on speakers' bureaus from Bristol‐Myers Squibb, and Ono Pharmaceutical. Y. Ohe has received research funding from MSD; and received honoraria from MSD. All remaining authors have declared no conflicts of interest.
